# Ferroptosis-Related IncRNAs Are Prognostic Biomarker of Overall Survival in Pancreatic Cancer Patients

**DOI:** 10.3389/fcell.2022.819724

**Published:** 2022-02-10

**Authors:** Dongjie Chen, Wenzhe Gao, Longjun Zang, Xianlin Zhang, Zheng Li, Hongwei Zhu, Xiao Yu

**Affiliations:** ^1^ Department of Hepatopancreatobiliary Surgery, The Third Xiangya Hospital, Central South University, Changsha, China; ^2^ Department of General Surgery, Affiliated Renhe Hospital of China Three Gorges University, Yichang, China

**Keywords:** pancreactic cancer, ferroptosis, long non coding RNA, prognostic biomarker, ceRNA network

## Abstract

Pancreatic cancer (PC) is one of the most lethal malignancies, the mortality and morbidity of which have been increasing over the past decade. Ferroptosis, a newly identified iron-dependent cell death pattern, can be induced by iron chelators and small lipophilic antioxidants. Nonetheless, the prognostic significance of ferroptosis-related lncRNAs in PC remains to be clarified. We obtained the lncRNA expression matrix and clinicopathological information of PC patients from The Cancer Genome Atlas (TCGA) and the International Cancer Genome Consortium (ICGC) datasets in the current study. Firstly, we conducted Pearson correlation analysis to delve into the ferroptosis-related lncRNAs, and univariate Cox analysis was implemented to examine the prognostic values in PC patients. Twenty-three prognostic ferroptosis-related lncRNAs were confirmed and loaded into the least absolute shrinkage and selection operator Cox (LASSO-Cox) analysis, then a ferroptosis-related lncRNA prognostic marker (Fe-LPM) was established in the TCGA dataset. Risk scores of patients were calculated and segregated PC patients into low-risk and high-risk subgroups in each dataset. The prognostic capability of Fe-LPM was also confirmed in the ICGC dataset. Gene set enrichment analysis (GSEA) revealed that several ferroptosis-related pathways were enriched in low-risk subgroups. Furthermore, we adopted a multivariate Cox regression to establish a nomogram based on risk score, age, pathological T stage and primary therapy outcome. A competing endogenous RNA (ceRNA) network was also created relied on four of the twenty-three ferroptosis-related lncRNAs. In conclusion, the eight Fe-LPM can be utilized to anticipate the overall survival (OS) of PC patients, which are meaningful to guiding clinical strategies in PC.

## Introduction

Pancreatic cancer (PC) is one kind of the most devastating carcinoma worldwide (Vincent et al., 2011). Although the therapeutic strategies of PC have been developed, widespread metastases and drug resistance remain to be challenges for clinicians. What’s more, the overlapping symptoms and long latency period hampered the early diagnosis of PC. These factors result in PC having a typically poor prognosis (Wolfgang et al., 2013). Hence, it is imperative to hunt for novel clinical regimens for PC patients (Dreyer et al., 2017; Neoptolemos et al., 2018).

Ferroptosis, a unique type of cell death distinct from other regulated cell death (RCD), plays a pivotal role in iron metabolism and lipid peroxidation (Zheng and Conrad, 2020; Tang et al., 2021). Since the first identification of ferroptosis in 2012, numerous studies have revealed that it can regulate the pathogenesis of various human malignancies (Stockwell et al., 2020). For instance, GPX4 overexpression or ACSL4 depletion in glioblastoma cells could promote tumor necrosis *via* neutrophil-induced ferroptosis (Yee et al., 2020), and CAFs were reported to inhibit ferroptosis in gastric cancer cells by targeting ALOX15 and blocking lipid-ROS accumulation (Zhang et al., 2020). Lately, some researchers have elucidated that the dysfunction of ferroptosis regulators is tightly associated with the progression of PC (Dai et al., 2020).

Long non-coding RNA (lncRNA) is a class of non-coding RNA whose length exceeds 200 nucleotides. With the deep recognition of lncRNAs, it is also believed that the aberrant expression and mutation of lncRNAs serve a crucial part in the malignant proliferation of PC (Xiong et al., 2017; Sun and Ma, 2019). For example, PLACT1 was previously reported to be an E2F1-mediated lncRNA promoting pancreatic ductal adenocarcinoma (PDAC) tumorigenicity and metastatic potential (Ren et al., 2020); THAP9-AS1 was considered as a potential biomarker to play an essential role in PDAC growth *via* enhancing YAP signaling (Li et al., 2020), and overexpression of GSTM3TV2 could strengthen the chemoresistance in PC (Xiong et al., 2019). However, few studies have focused on the mechanisms of how ferroptosis regulates the development and advance in lncRNA-mediated PC. On that account, fathoming out the relationship between ferroptosis of lncRNA and PC development is urgently needed to detect novel prognostic signatures that can behave as effective targets for treatment.

**FIGURE 1:**
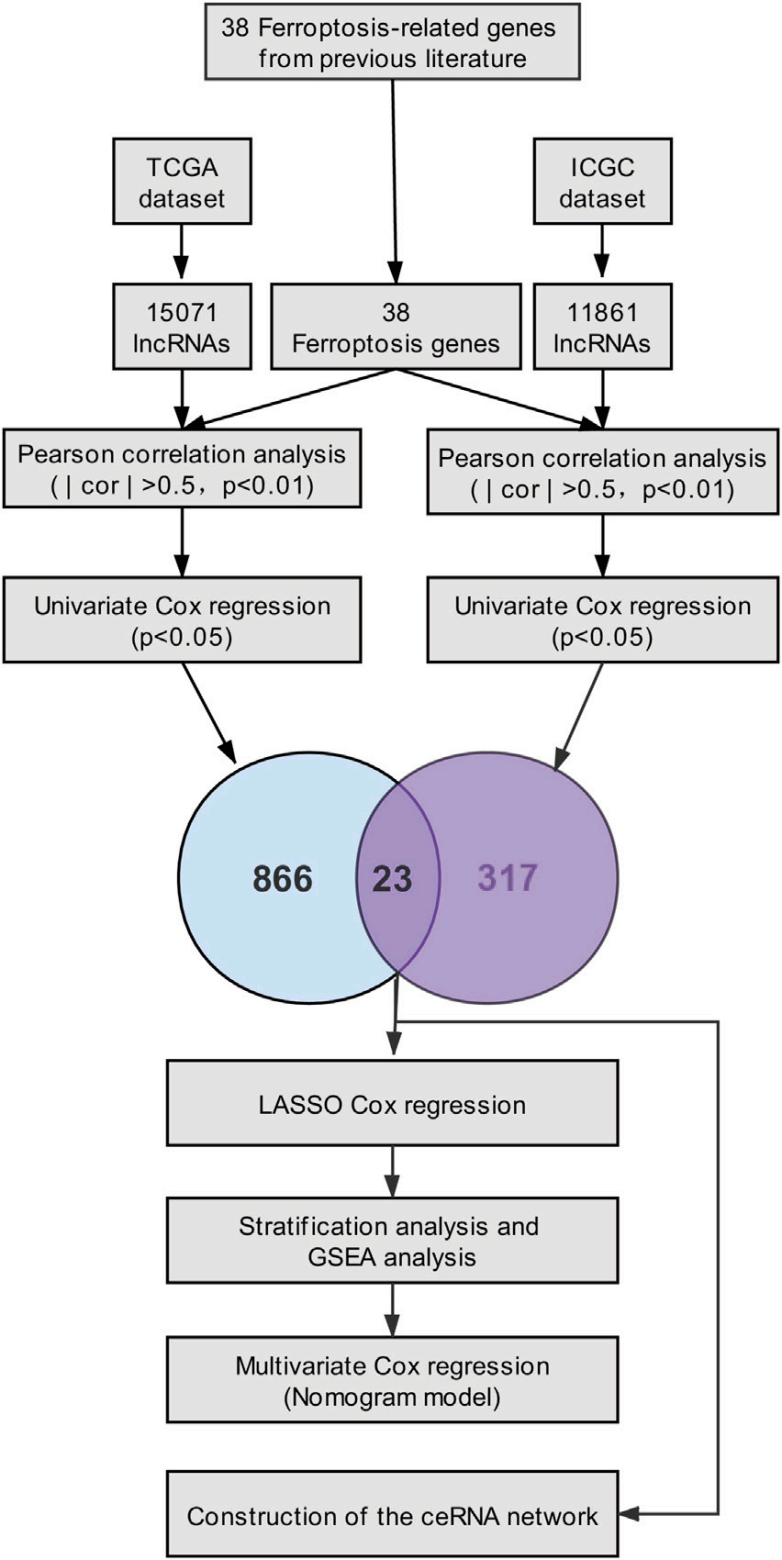
Flow diagram of the study.

In our study, based on the TCGA cohort and the ICGC cohort of PC patients, twenty-three prognostic ferroptosis-related lncRNAs were identified as prognostic ferroptosis-related lncRNAs. Then, we constructed a ferroptosis-related lncRNA prognostic marker (Fe-LPM) according to the predictive capacity of eight ferroptosis-related lncRNAs. In this way, PC patients were categorized as high-risk and low-risk subgroups, and we found many signal-transduction pathways converged on the low-risk subgroup according to GSEA analysis. Moreover, a competing risk nomogram model was established to predict the long-term survival of patients with PC. Last but not least, we constructed a ceRNA network to search the target miRNAs and mRNAs of these ferroptosis-related prognostic lncRNAs. The flowchart of the study was shown in Figure 1.

## Materials and Methods

### Data Processing

RNA-expression matrix and clinicopathological data of patients were extracted from the TCGA_PAAD (source: https://portal.gdc.cancer.gov/; *n* = 178) as a training set, and the data in ICGC_AU_PAAD (source: https://daco.icgc.org/; *n* = 91) were obtained from the ICGC website as a validation set. For ensuring the comparability between TCGA and ICGC datasets and removing the batch effect, TPM (Transcripts Per kilobase of exon model per Million mapped reads) data of RNA-expression matrix were log2 transformed and normalized *via* “limma” R package . Patients with absent prognostic information were excluded from our analysis. Additionally, based on previous literature (Stockwell et al., 2017; Hassannia et al., 2019), we retrieved the expression matrix of 38 ferroptosis-related genes from the TCGA and ICGC datasets.

### Generation and Assessment of the Ferroptosis-Related lncRNA Prognostic Marker

Aimed at identifying the ferroptosis-related prognostic lncRNAs, Pearson correlation analysis was first implemented (| cor | > 0.5, *p* < 0.01), and we performed univariate Cox analysis among TCGA and ICGC datasets separately. The ferroptosis-related lncRNAs extracted from two datasets were converged to seize the twenty-three prognostic ferroptosis-related lncRNAs. Afterward, The R package ‘‘glmnet’’ was used to conduct the LASSO-Cox analysis. LASSO model which performs both variable selection and regularization can shrink coefficients to zero. 10-time cross-validation was performed to access the optimal values for *λ*, and the minimum *λ* was selected. Then, we established a ferroptosis-related lncRNA prognostic marker (Fe-LPM) for the PC patient implicating eight ferroptosis-related lncRNAs. The formula of the risk score was constructed as follows:

$$\mathrm{Risk score}=\sum_{i=1}^{n}\mathrm{Coe}f_{i}*x_{i}$$

Where $Coef_{i}$ represents the coefficients, $x_{i}$ represents the normalized count of each ferroptosis-related lncRNAs. We performed the Principal component analysis (PCA) to check the identifiable capability of the Fe-LPM with the “gmodels” R package. Patients were divided into high-risk and low-risk subgroups on the threshold of median risk scores. Owing to analyzing the survival conditions of Fe-LPM, the optimal cut-off value was calculated using the R package “survminer”. The R package ‘‘timeROC’’ was applied to perform a time-dependent receiver operating characteristic (ROC) curve to estimate the forecasting capacity of the Fe-LPM. We conducted the univariate and multivariate Cox analyses to identify the independence of the Fe-LPM in predicting OS of PC patients. Based on the result of multivariate Cox regression, we developed a nomogram, and the calibration plot validated the robustness of the nomogram.

### Establishment of a Competing Endogenous RNA Network

For understanding the co-expression network of lncRNAs, miRNAs and mRNAs, four of twenty-three ferroptosis-related lncRNAs were picked to predict twenty-seven target miRNAs employing miRcode database. Then, we identified fifty-seven target mRNAs of twenty-seven miRNAs based on the MiRDB, miRTarBase, StarBase and TargetScan databases. The ceRNA network was visualized with ‘‘Cytoscape’’ software (version 3.8.2).

### Pathway Enrichment Analysis and Gene Set Enrichment Analysis Analysis

Based on the TCGA_PAAD training set, differential analysis was conducted, and then 1213 differentially expressed mRNAs (DEmRNAs) between high-risk and low-risk groups were selected according to the criteria of | log2(Fold Change) | > 1 and p < 0.05 utilizing the ‘‘DESeq2’’ R package. The 1213 DEmRNAs and the 57 target mRNAs in the ceRNA network were individually imported into the ‘‘Metascape’’ website for pathway enrichment analysis. Gene Set Enrichment Analysis (GSEA) was also performed to interrogate the potential pathways enriched in the high-risk and low-risk subgroups, using GSEA software (version 4.1.0). Gene expression data from the TCGA_PAAD cohort were loaded into GSEA, and the *c2.cp.kegg.v7.2.symbols.gmt* and *h.all.v7.2.symbols.gmt* were elected as the gene set database. The benchmark for which to be significantly enriched include nominal *p*-value < 0.05, normalized enrichment score (NES) > 1, and false discovery rate (FDR) *q*-value < 0.25.

### Statistical Analysis

The student’s *t*-test was used to compare the distribution of risk scores. Pearson correlation test was utilized to calculate the relationships between ferroptosis-related genes and lncRNAs. Chi-square test was performed to compare the categorical data of distinct groups. Wilcoxon test was used to compare ranked and independent data between different groups. Kaplan-Meier (KM) analysis with a log-rank test was used to compare the OS between different groups. All statistical analyses were performed using the R programming language (Version 4.0.3). Unless expressly stated, a *p*-value of less than 0.05 was deemed to be statistically significant.

## Results

### Identification of Ferroptosis-Related lncRNAs

**FIGURE 2:**
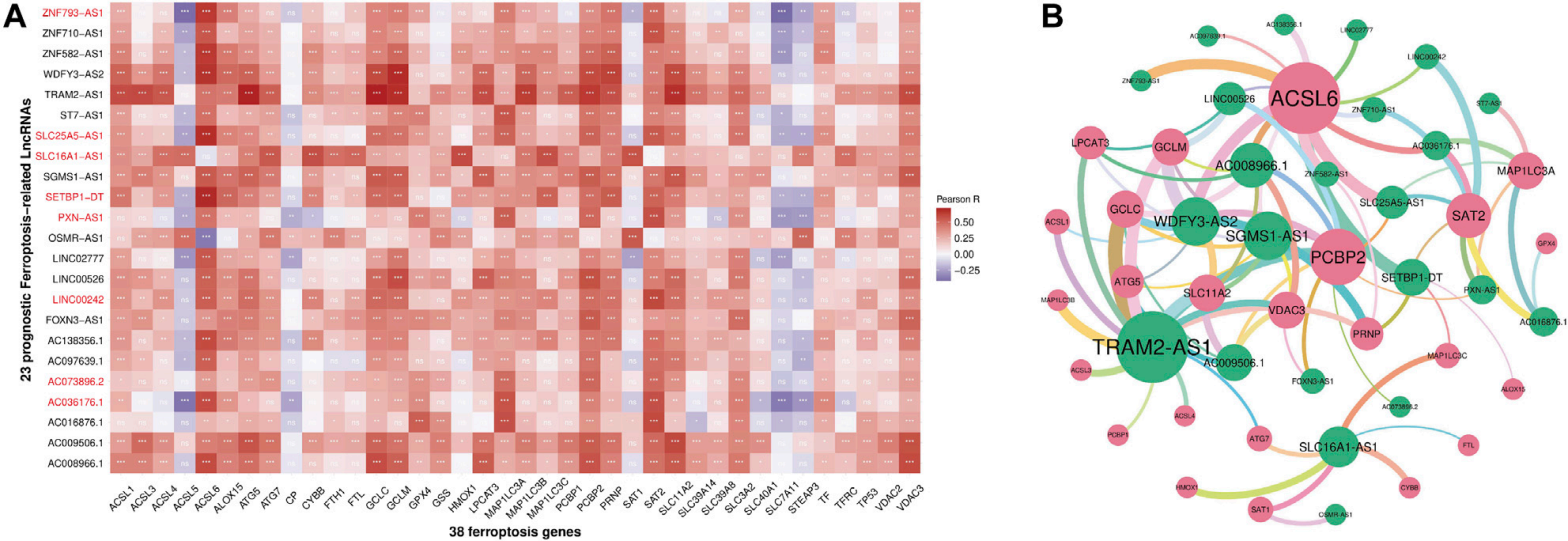
Heatmap **(A)** and correlation network **(B)** between 38 ferroptosis-related genes and 23 prognostic ferroptosis-related lncRNAs (ns: *p* ≥ 0.05, *p < 0.05, **p < 0.01, ***p < 0.001).

Through the ''GENCODE″ database, we pinpointed 15071 and 11861 lncRNAs in the TCGA and ICGC datasets separately. Then, we obtained the expression matrix of 38 ferroptosis-related genes from each dataset. Pearson correlation analysis was first carried out to explore ferroptosis-related lncRNAs, and thenceforth the univariate Cox regression was constructed to seek ferroptosis-related prognostic lncRNA (*p* < 0.05) in each dataset. Finally, we identified twenty-three ferroptosis-related lncRNAs, which dramatically correlated with the survival among both datasets. The association between 23 ferroptosis-related lncRNAs and 38 ferroptosis-related genes was displayed in Figure 2. The univariate Cox analysis of twenty-three ferroptosis-related lncRNAs was shown in Table 1.

**TABLE 1:** The 23 ferroptosis-related prognostic lncRNAs.

lncRNA	TCGA				ICGC			
	HR	HR.95L	HR.95H	*p*-value	HR	HR.95L	HR.95H	*p*-value
**OSMR-AS1**	**1.4915**	**1.0714**	**2.0763**	**0.0179**	**1.2615**	**1.0116**	**1.5732**	**0.0392**
**SLC16A1-AS1**	**1.4379**	**1.0252**	**2.0166**	**0.0354**	**1.2242**	**1.0028**	**1.4945**	**0.0469**
SETBP1-DT	0.8096	0.6639	0.9872	0.0369	0.7414	0.6194	0.8874	0.0011
AC097639.1	0.8032	0.6709	0.9617	0.0170	0.7965	0.6595	0.9621	0.0182
TRAM2-AS1	0.7956	0.6417	0.9863	0.0370	0.7370	0.5433	0.9998	0.0498
FOXN3-AS1	0.7658	0.6139	0.9552	0.0179	0.7542	0.5733	0.9921	0.0437
ZNF793-AS1	0.7639	0.6051	0.9645	0.0236	0.7920	0.6888	0.9107	0.0011
LINC02777	0.7626	0.5860	0.9923	0.0437	0.8125	0.6668	0.9900	0.0395
LINC00526	0.7624	0.5873	0.9898	0.0417	0.7570	0.6133	0.9345	0.0096
SLC25A5-AS1	0.7556	0.5987	0.9535	0.0182	0.6088	0.4681	0.7917	0.0002
ZNF582-AS1	0.7524	0.5734	0.9871	0.0400	0.8326	0.7038	0.9850	0.0326
AC073896.2	0.7519	0.5795	0.9755	0.0318	0.5455	0.3846	0.7736	0.0007
AC009506.1	0.7479	0.5960	0.9386	0.0122	0.6740	0.4885	0.9298	0.0163
SGMS1-AS1	0.7370	0.5463	0.9942	0.0457	0.7119	0.5489	0.9233	0.0104
AC138356.1	0.7351	0.5439	0.9936	0.0453	0.7396	0.5989	0.9135	0.0051
AC008966.1	0.7348	0.5746	0.9397	0.0140	0.7491	0.5969	0.9402	0.0127
LINC00242	0.7280	0.5712	0.9279	0.0103	0.6744	0.5436	0.8366	0.0003
ST7-AS1	0.7146	0.5571	0.9167	0.0082	0.7633	0.6110	0.9534	0.0173
WDFY3-AS2	0.7067	0.5573	0.8961	0.0042	0.7835	0.6359	0.9655	0.0221
ZNF710-AS1	0.6972	0.5354	0.9079	0.0074	0.7130	0.5704	0.8912	0.0030
PXN-AS1	0.6910	0.5026	0.9501	0.0229	0.6138	0.4550	0.8279	0.0014
AC036176.1	0.6817	0.5213	0.8915	0.0051	0.6414	0.5054	0.8140	0.0003
AC016876.1	0.6378	0.4634	0.8778	0.0058	0.6581	0.4901	0.8838	0.0054

lncRNAs marked with bold font were risky lncRNAs and others were protective lncRNAs.

### Construction of the Ferroptosis-Related lncRNA Prognostic Marker in the The Cancer Genome Atlas Dataset

**FIGURE 3:**
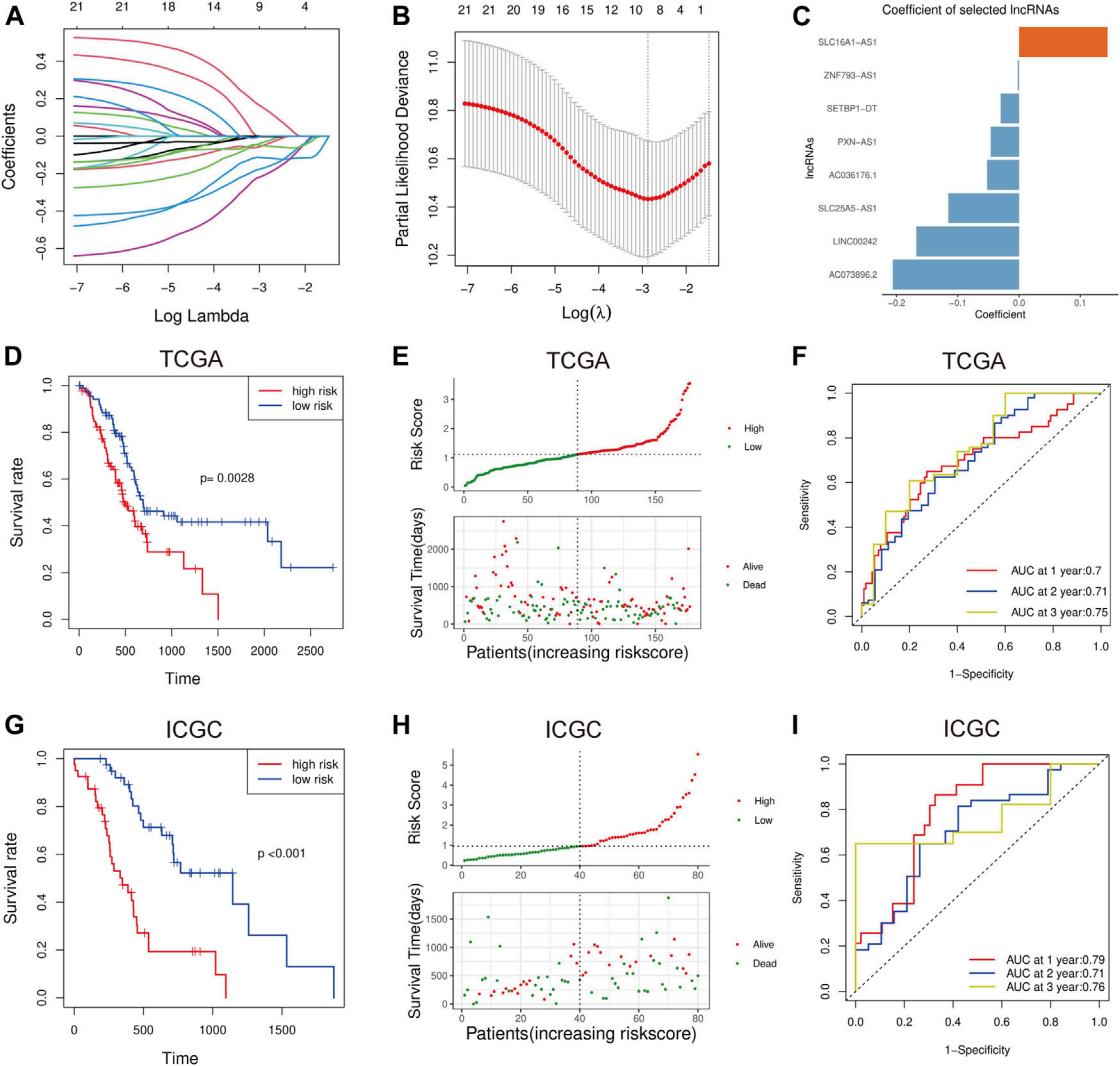
The least absolute shrinkage and selection operator Cox (LASSO-Cox) analysis was implemented **(A, B)**, and the coefficients were calculated by the 10-fold cross-validation based on minimum criteria **(C)**. Kaplan-Meier analysis suggests the OS in the high-risk and low-risk groups in the TCGA cohort **(D)**. Distribution of risk scores and survival status of PC patients in the TCGA cohort **(E)**. Receiver operating characteristic (ROC) curve analysis for Fe-LPM in the TCGA cohort **(F)**. In the ICGC validation cohort, the results of the Kaplan-Meier analysis were consistent with those in the TCGA cohort **(G)**. Distribution of risk scores and survival status of PC patients in the ICGC cohort **(H)**. Receiver operating characteristic (ROC) curve analysis for Fe-LPM in the ICGC validation cohort **(I)**.

The LASSO-Cox analysis was performed using the expression matrix of the twenty-three ferroptosis-related prognostic lncRNA mentioned above. The Fe-LPM encompassed eight ferroptosis-related lncRNAs in the TCGA dataset was identified (Figures 3A,B), the coefficient of the Fe-LPM was shown in Figure 3C. Based on TCGA dataset, a risk score was computed mentioned in the Materials and Methods part, and patients were then split for survival analysis into high-risk and low-risk subgroups on the threshold of median risk scores. The principal component analysis (PCA) indicated that we obtain a high degree of discrimination between high-risk and low-risk subgroups in the TCGA cohort (Supplementary Figures S1A, B), showing a remarkable difference between these different subgroups. KM survival curves considered that patients from the high-risk subgroup had considerably worse clinical outcomes than those from the low-risk subgroup (Figure 3D). The distribution of the risk score was shown in Figure 3E. ROC curves appraised the forecasting capability of the Fe-LPM for overall survival, and the area under the curve (AUC) reached 0.70 at 1 year, 0.71 at 2 years, 0.75 at 3 years (Figure 3F).

### Validation of the Ferroptosis-Related lncRNA Prognostic Marker in the International Cancer Genome Consortium Dataset

To verify the stability of the prognostic markers constructed in the TCGA dataset, patients in the ICGC validation set were also assigned into high-risk and low-risk subgroups relative to the median risk scores calculated with the same formula as that from the training set. PCA in ICGC datasets was shown in Supplementary Figures S1C, D. Similar to the conclusion in the TCGA cohort, patients with high-risk scores had shorter OS than the low-risk ones in the ICGC dataset (Figure 3G). The distribution of the risk score was shown in Figure 3H. The AUC of the Fe-LPM in the ICGC cohort was 0.79 at 1 year, 0.71 at 2 years, 0.76 at 3 years (Figure 3I).

### Prognostic Analysis of the Eight Ferroptosis-Related lncRNA Prognostic Marker

**FIGURE 4:**
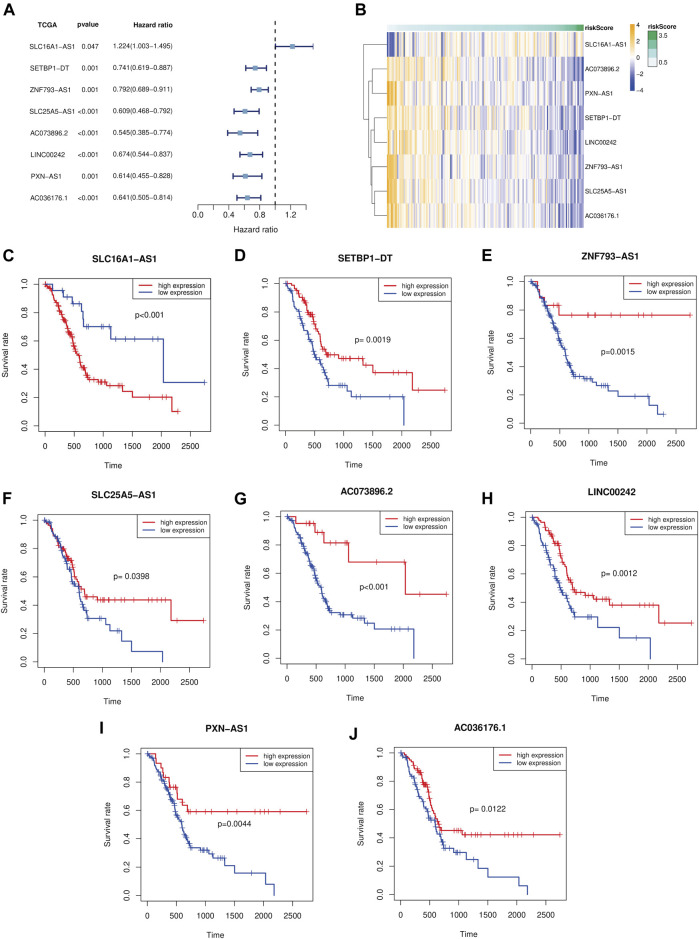
Forest plot of the univariate Cox analysis in eight Fe-LPM **(A)**. Heatmap showed the relationship between risk score and expression of the eight Fe-LPM **(B)**. Kaplan-Meier analysis in different expression subgroups of the eight Fe-LPM **(C–J)**.

We implemented the univariate Cox analysis to estimate the prognostic role of the eight Fe-LPM. The result indicates that SLC16A1-AS1 is a risk factor with HR > 1, whereas SETBP1-DT, ZNF93-AS1, SLC25A5-AS1, AC073896.2, LINC00242, PXN-AS1 and AC036176.1 are protective factors (Figure 4A). The heatmap exhibits that SLC16A1-AS1 expression increased with increasing risk score, while SETBP1-DT, ZNF93-AS1, SLC25A5-AS1, AC073896.2, LINC00242, PXN-AS1 and AC036176.1 expression decreased with increasing risk score (Figure 4B). The Kaplan-Meier curve certified that lower expression of SLC16A1-AS1 and higher expression of SETBP1-DT, ZNF93-AS1, SLC25A5-AS1, AC073896.2, LINC00242, PXN-AS1 and AC036176.1 corresponded with higher survival time in the TCGA cohort (Figures 4C–J).

### Clinical Relevance of the Ferroptosis-Related lncRNA Prognostic Marker

**FIGURE 5:**
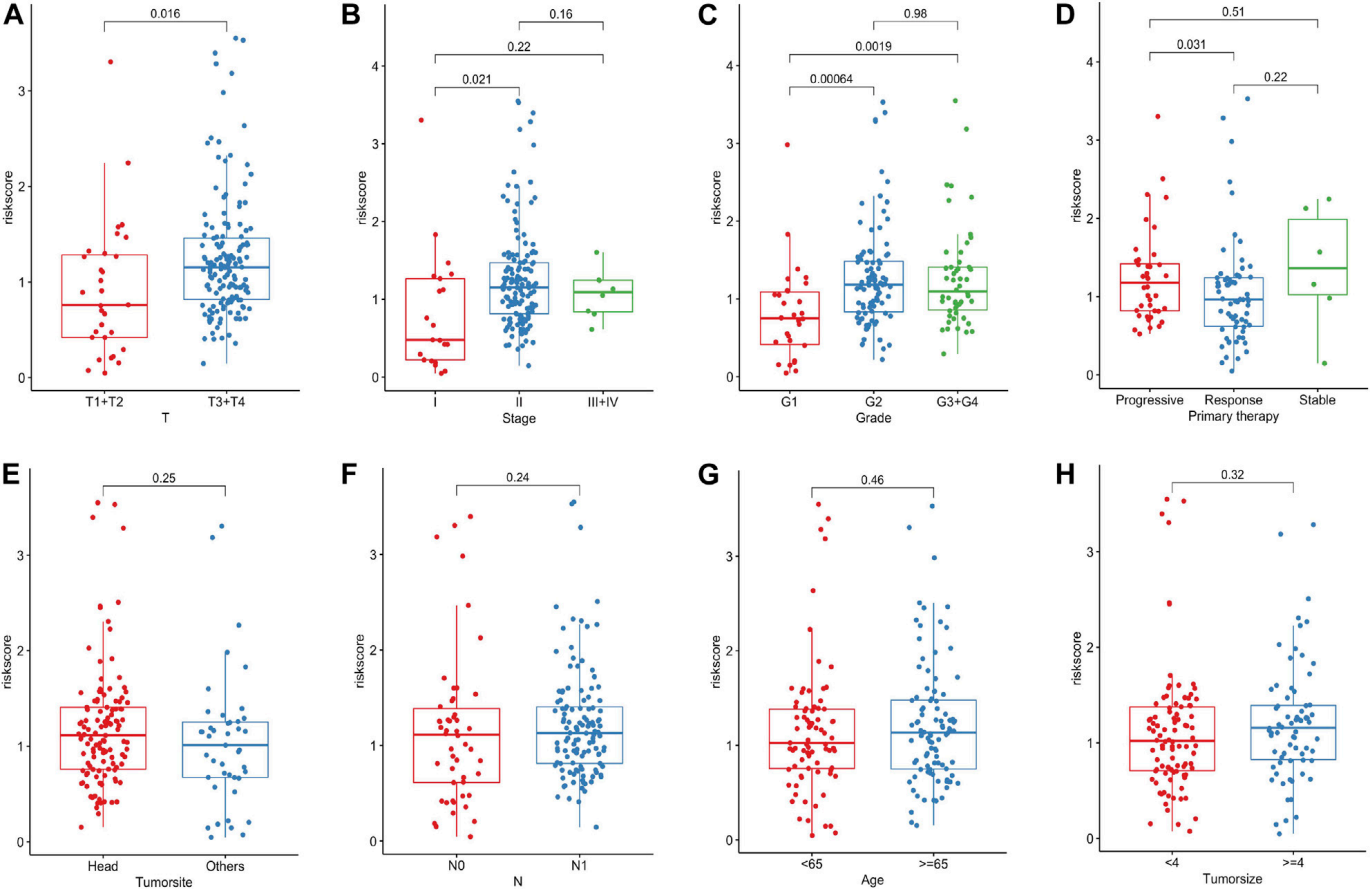
The risk score significantly correlated with pathological T stage, TNM stage, grade and primary therapy outcome of PC patients **(A–D)**. The risk score was not related to the tumor site, pathological N stage, age and tumor size **(E–H)**.

To clarify the associations between risk scores and clinicopathological characteristics, we further scrutinize the clinicopathological information from the TCGA cohort. The result manifested that the risk score apparently corresponded with pathological T stage, TNM stage, grade and primary therapy outcome of PC patients (Figures 5A–D). The stronger the tendency to higher pathological T stage, the higher the risk scores, which may explain the poorer survival rate of the high-risk group. Consistently, we found that risk scores were higher in advanced PC than those in early TNM stages and grades. Furthermore, patients with progressive status after primary therapy obtained higher risk scores than those with responsive status, suggesting that this Fe-LPM may serve as a potential evaluation index of primary therapy in PC patients. In contrast, the tumor site, pathological N stage, age and tumor size proved nonsensical (Figures 5E–H).

### Pathway Enrichment Analysis and Gene Set Enrichment Analysis Analysis in the The Cancer Genome Atlas Dataset

**FIGURE 6:**
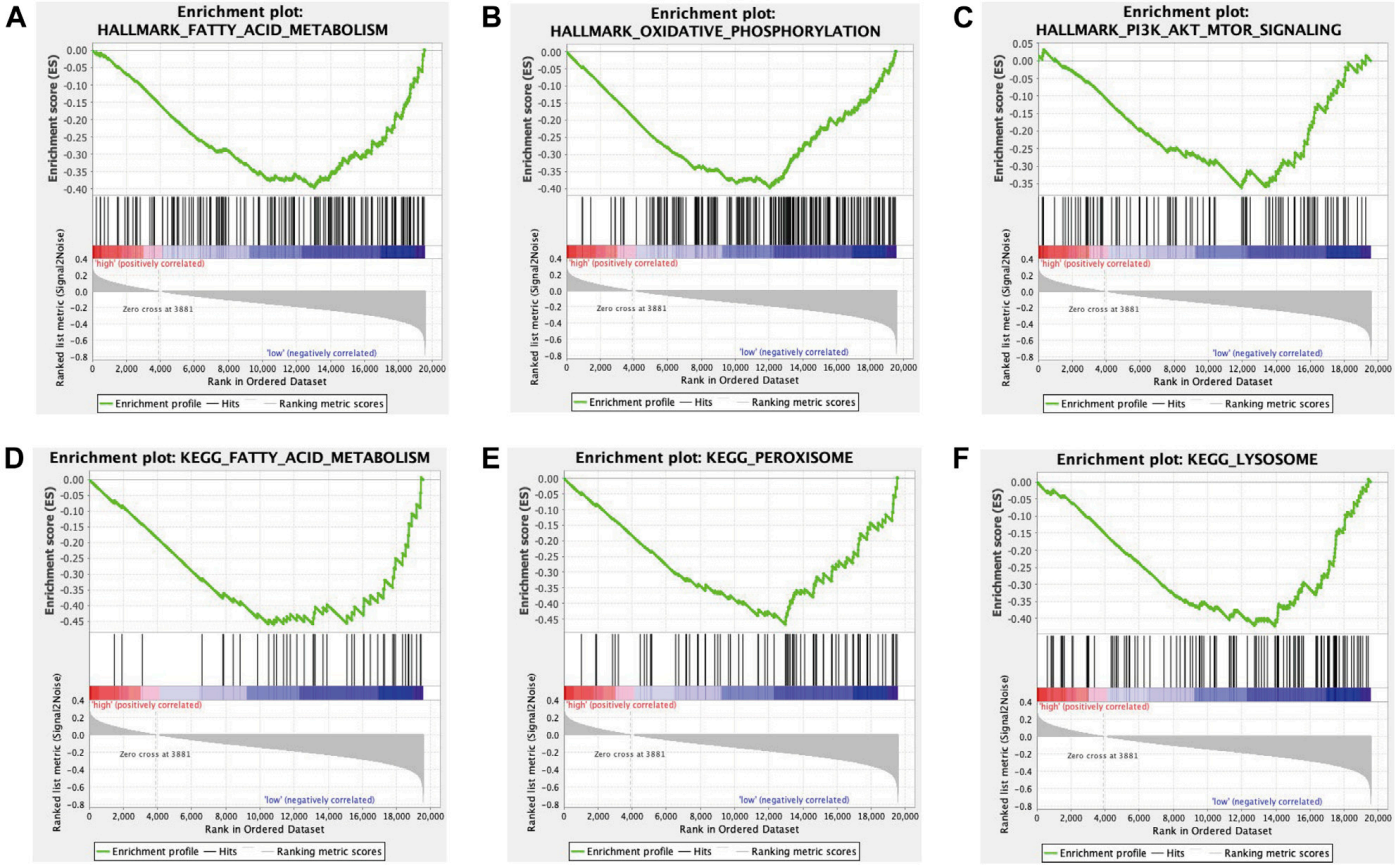
GSEA illustrated that genes in the low-risk subgroup were significantly enriched in fatty acid metabolism **(A, D)**, oxidative phosphorylation **(B)**, PI3K-AKT-mTOR signaling **(C)**, peroxisome **(E)** and lysosome **(F)**.

For exploring the underlying pathway among the high-risk and low-risk subgroups, 1213 differentially expressed mRNAs (DEmRNAs) between high-risk and low-risk groups were selected according to the criteria of | log_2_(Fold change) | > 1 and *p* < 0.05. These DEmRNAs were significantly enriched in chemical synaptic transmission, regulation of hormone levels, presynapse and regulation of ion transport (Supplementary Figure S2). The results of GSEA revealed that the genes in the low-risk subgroup were significantly enriched in fatty acid metabolism, peroxisome, oxidative phosphorylation, PI3K-AKT-mTOR signaling and lysosome (Figures 6A–F).

### The Independence of the Ferroptosis-Related lncRNA Prognostic Marker in Predicting Overall Survival in Pancreatic Cancer

**FIGURE 7:**
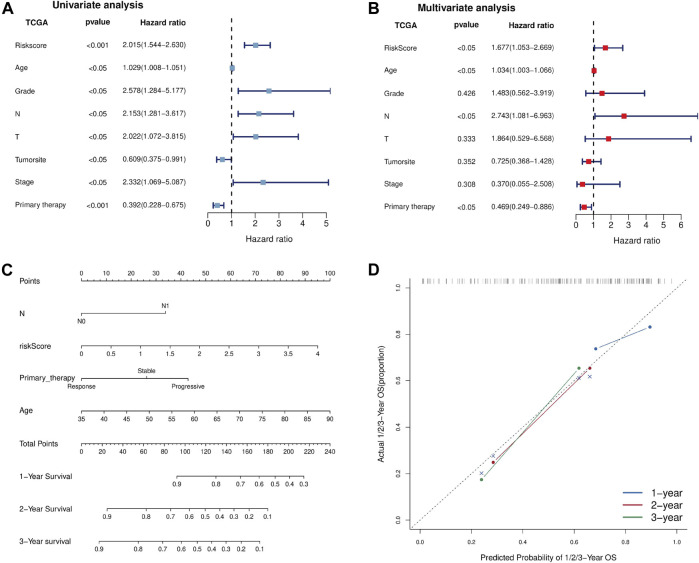
Univariate and multivariate Cox analyses were performed based on risk scores and other clinical features in the TCGA dataset **(A, B)**. A nomogram was constructed based on pathological T stage, risk score, primary therapy outcome and age in the TCGA cohort **(C)**. A calibration plot confirmed the robustness of the Fe-LPM **(D)**.

To evaluate whether the Fe-LPM was an independent prognostic indicator for OS, univariate and multivariate Cox regression analyses were implemented. In the TCGA dataset, both univariate Cox analysis and multivariate Cox analysis showed that Fe-LPM was markedly correlated with survival status (Figures 7A,B). To establish a predictive tool for quantitative analysis of OS in PC patients, we initiated a prognostic nomogram based on the pathological T stage, risk score, primary therapy outcome and age in the TCGA cohort (Figure 7C). Calibration plots showed that the predictive concordance of this nomogram (Figure 7D).

### Construction of the Competing Endogenous RNA Regulatory Network

**FIGURE 8:**
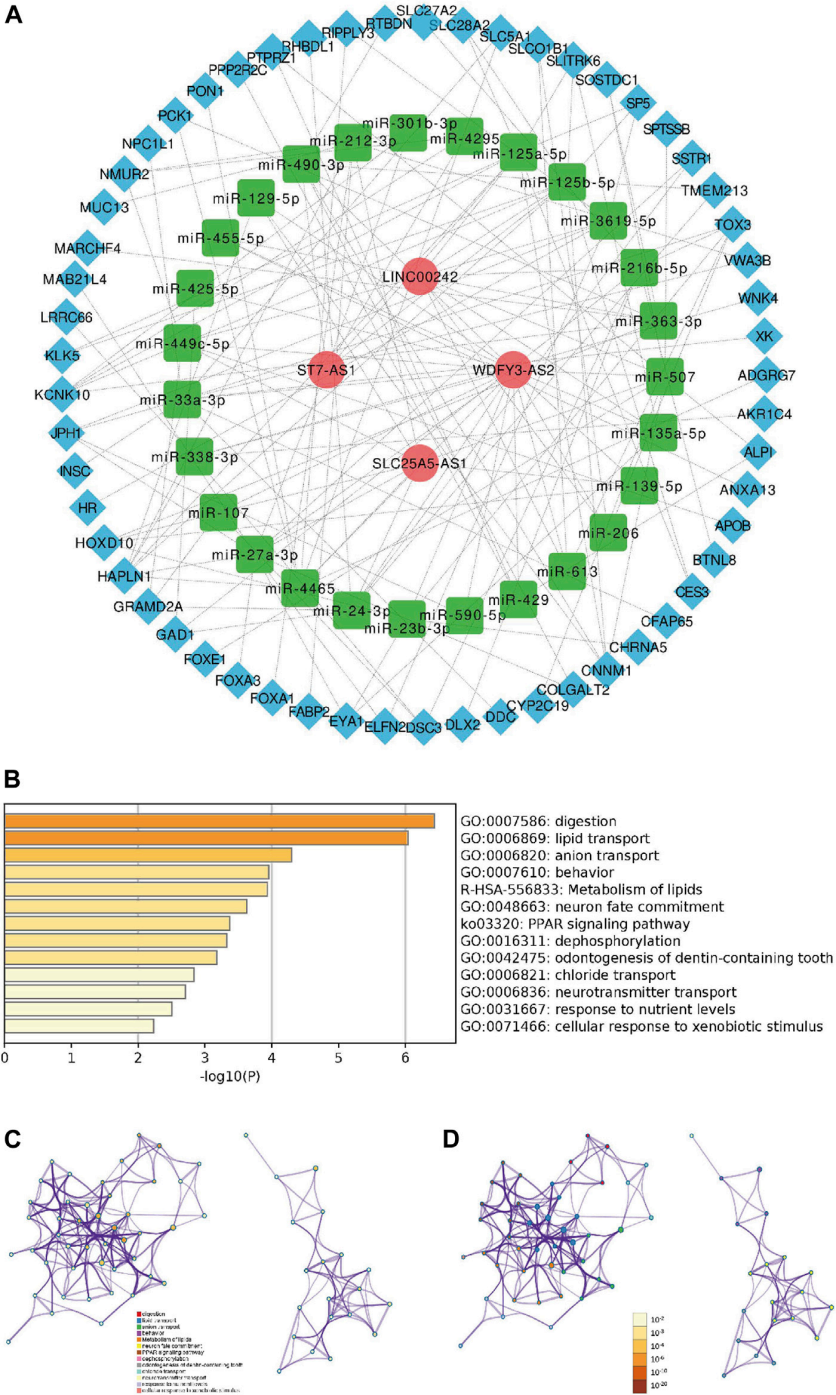
A ceRNA network including 4 lncRNAs-27 miRNAs-57 mRNAs was established **(A)**. The heatmap **(B),** cluster ID network **(C)** and *p*-value network **(D)** of enriched pathways based on 57 target mRNAs.

The ceRNA network comprising lncRNAs, miRNAs and mRNAs was constructed for the sake of systematically probing into the potential regulatory mechanism. According to those mentioned above twenty-three ferroptosis-related lncRNAs, four of twenty-three lncRNAs were filtered from the miRcode database, and twenty-seven target miRNAs were distinguished. Then we use the MiRDB, miRTarBase, StarBase and TargetScan databases to extracted fifty-seven target mRNAs on account of twenty-seven miRNAs. At length, four lncRNAs, twenty-seven miRNAs and fifty-seven mRNAs were included in the ceRNA network (Figure 8A, Supplementary Figure S3). The pathway enrichment analysis of fifty-seven target mRNAs elucidated that these mRNAs were enriched in lipid transport, anion transport and dephosphorylation (Figures 8B–D), suggesting that the Fe-LPM was definitely correlated with the process of ferroptosis.

## Discussion

Ferroptosis, an extraordinary cell death pathway driven by iron-dependent lipid peroxidation, regulates the epigenetic program and metabolic changes in numerous cancers (Wu et al., 2020). Multiple studies have already shown that specific lncRNAs can alleviate the malignancy of tumors by regulating the ferroptosis-related pathway. Interacted with G3BP1, the lncRNA P53RRA could restrain lung cancer progression *via* activating the p53 signaling pathway which correlated with ferroptosis (Mao et al., 2018). LncRNA GABPB1-AS1 inhibits the translation of GABPB1, causing the accumulation of iron-dependent reactive oxygen species (ROS) and improving the overall survival of HCC patients (Qi et al., 2019). Additionally, most studies have confirmed that lncRNAs might function as ceRNA of mRNAs, thereby manipulating ferroptosis-mediated tumor progression. LINC00336 functions as an oncogene in lung cancer and regulates cystathionine-β-synthase (CBS) expression by competing for miR6852 (Wang et al., 2019). The lncRNA MT1DP could sensitize NSCLC cells to erastin-induced ferroptosis by regulating the miR-365a-3p/NRF2 axis, mitigating the mortality of NSCLC (Gai et al., 2020). After pondering these clues, we hypothesize that ferroptosis is tightly linked to lncRNAs, and we ought to concentrate on the potential interaction between lncRNAs and ferroptosis to uncover underlying prognostic biomarkers.

In the aggregate, 257 PC patients were involved in detecting the prognostic value of ferroptosis-related lncRNAs. Twenty-three ferroptosis-related prognostic lncRNAs were identified, and eight of them were singled out as the Fe-LPM. LNC00242 was high-expressed in gastric cancer (GC) tissue regulated by LINC00242/miR-1-3p/G6PD axis (Deng et al., 2021), and it may be a promising biomarker of GC (Zhong et al., 2020). Mediated by E2F1, SLC16A1-AS1 could progress bladder cancer to the invasive stage (Logotheti et al., 2020) and serve as a new diagnostic indicator in hepatocellular carcinoma (Song et al., 2019; Pei et al., 2020). It has been reported that SLC25A5-AS1 functions as a suppressor in the progression of gastric cancer to regulate cellular behaviors *via* the miR-19a-3p/PTEN/PI3K/AKT signaling pathway (Li et al., 2019). PXN-AS1 stifles PC progression by serving as a sponge to suppress the expression of miR-3064 (Yan et al., 2019) and it also plays vital roles in hepatocarcinogenesis as a prognostic biomarker (Yuan et al., 2017). Since half of the eight lncRNAs were reported to be associated with tumor progression, the others need to be further determined their mechanism in the process of ferroptosis by experiments in our future studies.

According to the median risk score calculated by the coefficient of eight Fe-LPM, the PC patients were stratified into the high-risk and low-risk subgroups. To further investigate the biological process and pathway between the two subgroups, GSEA revealed fatty acid metabolism, peroxisome, oxidative phosphorylation, PI3K-AKT-mTOR signaling and lysosome were enormously enriched in the low-risk subgroup. These signaling pathways have been confirmed to be indispensable for ferroptosis (Stockwell et al., 2017), implying that the low-risk subgroup is more sensitive to ferroptosis. Polyunsaturated fatty acid (PUFA) biosynthesis pathway has been reported to be a marker for predicting the ability of ferroptosis-mediated therapy in gastric cancer (Lee et al., 2020). Peroxisome and oxidative phosphorylation also participate in the biological process of ferroptosis (Tang and Kroemer, 2020; Yusuf et al., 2020). Mutation of PI3K-AKT-mTOR signaling protects cancer cells from ferroptosis death through SREBP1/SCD1-mediated lipogenesis (Yi et al., 2020). Besides, various studies have reported that ferroptosis can mitigate gemcitabine resistance and lead to a favorable prognosis in pancreatic cancer cells (Ye et al., 2020; Ye et al., 2021), which is consistent with the result of our study: most of the Fe-LPM are protective factors based on the univariate Cox regression analysis.

Predicting clinical outcome endpoints is necessary for personalized and translational medicine (Behrens et al., 2017). Although numerous efforts have been exerted to research novel drugs that can suppress the PC, we can only expect to successfully translate genomic findings into clinical practice through the development of genome-sequencing technology (Singh et al., 2019). Consequently, we made further efforts to explore the prognostic value of the Fe-LPM. The univariate and multivariate Cox analyses identified that Fe-LPM was an independent indicator for OS. A nomogram was based on four clinicopathological features (pathological T stage, TNM stage, grade and primary therapy outcome of PC patients) was constructed and validated by calibration plot. Afterward, a ceRNA network was established including four lncRNAs, twenty-seven miRNAs and fifty-seven mRNAs. The above results confirmed that Fe-LPM may propose new prospects and intervention measures for the treatment of PC and provides a robust theoretical basis for future clinical judgments.

Given all the results offered above, we concluded that the eight Fe-LPM (including SLC16A1-AS1, SETBP1-DT, ZNF93-AS1, SLC25A5-AS1, AC073896.2, LINC00242, PXN-AS1 and AC036176) prove to be a prognostic biomarker of OS for PC patients. Function prediction further confirmed the importance of Fe-LPM in PC, offering us a comparatively systematic and comprehensive knowledge of the links among ferroptosis, lncRNAs and PC. However, it is observed that the shortage of clinical information in ICGC_AU_PAAD leads to a lack of profound validations in the ICGC cohort. On the other hand, our study should make further validations *in vivo* and vitro soon.

## Data Availability

The datasets presented in this study can be found in online repositories. The names of the repository/repositories and accession number(s) can be found in the article/Supplementary Material.
