# Supplementary material for: Ferroptosis-Related IncRNAs Are Prognostic Biomarker of Overall Survival in Pancreatic Cancer Patients
Source: Front Cell Dev Biol. 2022 Feb 10;10:819724. doi: 10.3389/fcell.2022.819724 (PMC8866714; doi:10.3389/fcell.2022.819724)
Supplement: Supplementary file 1 [file DataSheet1.PDF]

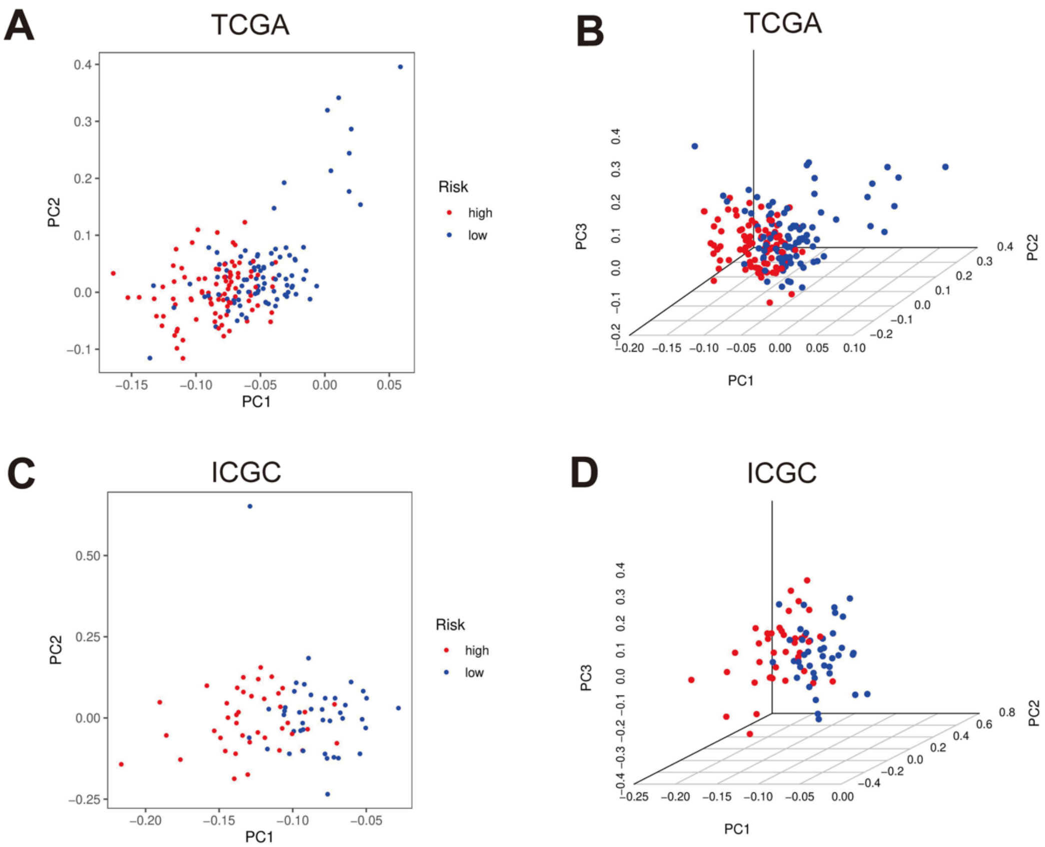

Supplementary Figure 1. The 2D **(A, B)** and 3D **(C, D)** plot of PCA based on the expression matrix of the 8 Fe-LPM in TCGA and ICGC dataset. These considered that we do obtain a high degree of discrimination between low-risk and high-risk subgroup.

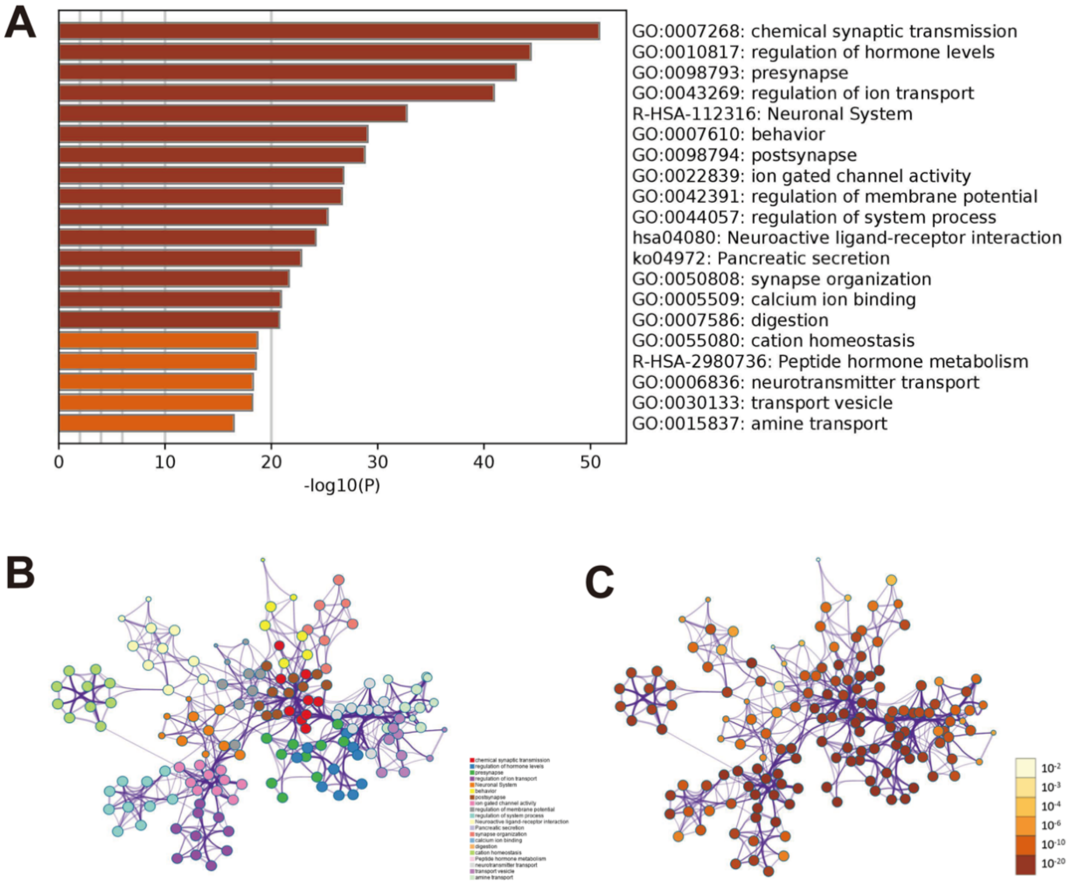

Supplementary Figure 2. Pathway enrichment analysis between the low-risk and high-risk subgroups was performed in TCGA dataset. The barpolt **(A)**, cluster ID network **(B)** and p-value network **(C)** of enriched pathways based on 8049 differentially expressed genes (DEGs).

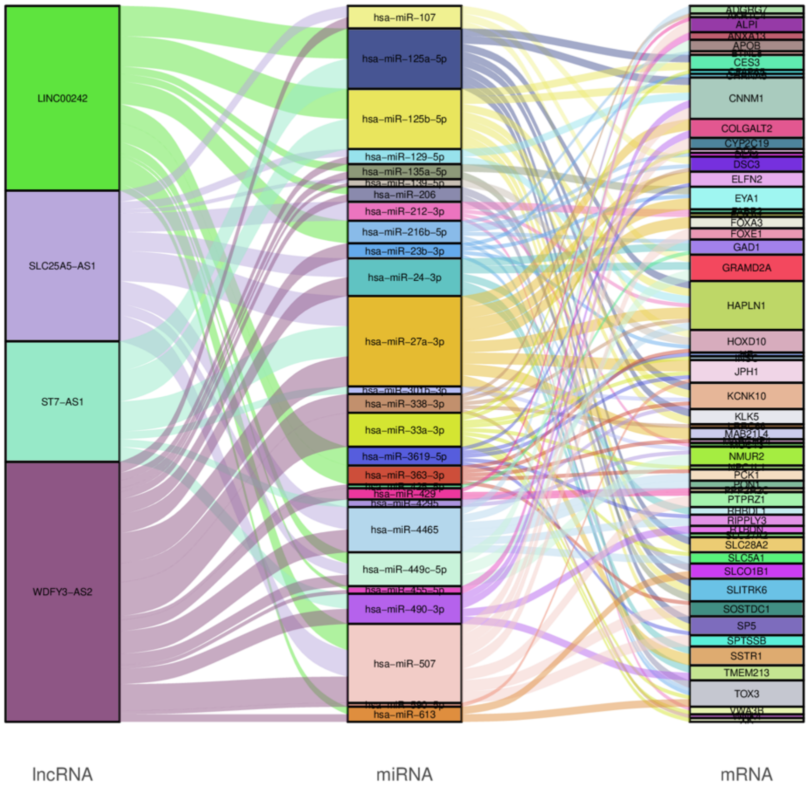

Supplementary Figure 3. Sankey plot of the ceRNA network, showing the relationship among 4 lncRNAs-27 miRNAs-57 mRNAs.
